# Serial change of C1 inhibitor in patients with sepsis: a preliminary report

**DOI:** 10.1186/cc14114

**Published:** 2015-03-16

**Authors:** T Hirose, H Ogura, K Jinkoo, Y Nakamura, H Hosotsubo, T Shimazu, E Kitano, M Hatanaka

**Affiliations:** 1 Osaka University Graduate School of Medicine, Suita, Japan; 2 Kobe Tokiwa University, Kobe, Japan

## Introduction

C1 inhibitor (C1INH), belonging to the superfamily of serine protease inhibitors, regulates not only complement system, but also the plasma kallikrein-kinin system, fibrinolytic system and coagulation system. The biologic activities of C1INH can be divided into the regulation of vascular permeability and anti-inflammatory functions. In recent years, hereditary angioedema (HAE), caused by an inherited deficiency of C1INH, has been focused. During HAE attacks, vascular permeability was markedly increased, which leads to angioedema. In sepsis, significant endothelial hyperpermeability is similarly observed systemically, but the role of C1INH has not been clarified in the pathogenesis. The serial change of C1INH in patients with sepsis is not clear. The objective of this study was to clarify the serial change in C1INH in patients with sepsis and evaluate the impact of C1INH on their clinical course.

## Methods

We serially examined C1INH activity values (normal range 70 to 130%) and quantitative values (normal range 160 to 330 μg/ml) in patients with sepsis during the period between December 2012 and February 2013. We also analyzed their clinical course: prognosis, volume of infusion, body weight, urine volume, catecholamine administration, and steroid administration.

## Results

The serial change of C1INH was evaluated in five patients with sepsis (three male and two female; four survivors and one nonsurvivor; mean age, 68 ± 11 years). In the nonsurvivor, C1INH activity on admission value was 97.2% (normal range), and quantitative value was 133.1 μg/ml (below normal). In the patient with severe sepsis requiring fluid resuscitation, catecholamine and steroid administration to maintain hemodynamics, C1INH activity value on admission was 94.4% (normal range), and quantitative value was 126.7 μg/ml (below normal range). His general condition was improved on day 6, and C1INH activity value and quantitative value increased (139.9%; above normal range, 250.1 μg/ml; normal range). In the other three patients with sepsis not requiring steroid administration, C1INH activity value on admission was 130.6 ± 8.7% (above normal range), and quantitative value was 215 ± 26.5 μg/ml (normal range).

## Conclusion

In the nonsurvivor or the severe patient with sepsis requiring steroid administration, the enhancement of C1INH activity was not observed, and the C1INH quantitative values were low. Further evaluation of the serial change of C1INH and the validity of C1INH replacement therapy in patients with septic shock may lead to a new strategy for management in sepsis.

